# Long term follow-up of cervical intervertebral disc herniation inpatients treated with integrated complementary and alternative medicine: a prospective case series observational study

**DOI:** 10.1186/s12906-016-1034-z

**Published:** 2016-02-04

**Authors:** Sang Hyun Baek, Jae Woo Oh, Joon-Shik Shin, Jinho Lee, Yoon Jae Lee, Me-riong Kim, Yong-jun Ahn, Areum Choi, Ki Byung Park, Byung-Cheul Shin, Myeong Soo Lee, In-Hyuk Ha

**Affiliations:** 1 Jaseng Spine and Joint Research Institute, Jaseng Medical Foundation, Seoul, Republic of Korea; 2 Division of Clinical Medicine, School of Korean Medicine, Pusan National University, Yangsan, Republic of Korea; 3 Medical Research Division, Korea Institute of Oriental Medicine, Daejeon, Republic of Korea

**Keywords:** Cervical intervertebral disc herniation, Complementary and alternative medicine, Integrative treatment, Inpatient treatment

## Abstract

**Background:**

Symptomatic cervical intervertebral disc herniation (IDH) presenting as neck pain accompanied by arm pain is a common affliction whose prevalence continues to rise, and is a frequent reason for integrative inpatient care using complementary and alternative medicine (CAM) in Korea. However, studies on its long term effects are scarce.

**Methods:**

A total 165 patients with cervical IDH admitted between January 2011 and September 2014 to a hospital that provides conventional and Korean medicine integrative treatment with CAM as the main modality were observed in a prospective observational study. Patients underwent CAM treatment administered by Korean medicine doctors (KMDs) in accordance with a predetermined protocol for the length of hospital stay, and additional conventional treatment by medical doctors (MDs) as referred by KMDs. Short term outcomes were assessed at discharge and long term follow-ups were conducted through phone interviews after discharge. Numeric rating scale (NRS) of neck and radiating arm pain, neck disability index (NDI), 5-point patient global impression of change (PGIC), and factors influencing long term satisfaction rates in PGIC were assessed.

**Results:**

Of 165 patients who received inpatient treatment 20.8 ± 11.2 days, 117 completed the long term follow-up up at 625.36 ± 196.7 days post-admission. Difference in NRS between admission and discharge in the long term follow-up group (*n* = 117) was 2.71 (95 % CI, 2.33, 3.09) for neck pain, 2.33 (95 % CI, 1.9, 2.77) for arm pain, and that of NDI 14.6 (95 % CI, 11.89, 17.32), and corresponding scores in the non-long term follow-up group (*n* = 48) were 2.83 (95 % CI, 2.22, 3.45) for neck pain, 2.48 (95 % CI, 1.84, 3.12) for arm pain, and that of NDI was 14.86 (95 % CI, 10.41, 19.3). Difference in long term NRS of neck pain and arm pain from baseline was 3.15 (95 % CI, 2.67, 3.64), and 2.64 (95 % CI, 1.99, 3.29), respectively. PGIC was reported to be “satisfactory” or higher in 79.5 % of patients at long term follow-up.

**Conclusions:**

Though the observational nature of this study limits us from drawing a more decisive conclusion, these results suggest that integrative treatment focused on CAM in cervical IDH inpatients may achieve favorable results in pain and functional improvement.

**Trial registration:**

ClinicalTrials.gov Identifier: NCT02257723. Registered October 2, 2014.

## Background

Neck pain is a common compliant whose point prevalence is estimated at 10–18 %, with lifetime prevalence reaching 30–50 %. Prevalence of neck pain in populations aged 40 or older is approximately 20 % [1, 2]. Neck pain is also related with restricted neck movement [3], and frequently accompanied by headache, dizziness, visual impairment, tinnitus, and autonomic nervous system dysfunction [4, 5]. Frequent concurrent symptoms include upper extremity pain and neurological disorders [6], and neck pain symptoms also persist in many cases leading to work loss due to discomfort [7]. Neck-related disability is generally more serious in patients with radiating pain than pain limited to the neck area [8, 9], and the main characteristic of cervical intervertebral disc herniation (IDH) is arm pain in the region innervated at the herniated disc level and/or compressed nerve root [10, 11].

The range of available treatments for cervical IDH is vast, spanning conservative treatments to various surgical modalities. Conservative treatments include NSAIDs, oral steroids, steroid injections, patient education, rest, Thomas collars, and physical therapy [12–14]. Surgical treatment may be considered when conservative treatment fails. Neuropathy from spinal cord compression is an absolute indication for surgery. Other indications include nerve root compression signs and related motor and sensory loss. Relative indications may involve decreased quality of life due to prolonged chronic pain [15]. While surgical treatment may benefit some patients suffering severe neurological symptoms, most studies on neuropathic pain of the spine state that the long term effects are not significant [16–20]. Although studies on the effect of conservative treatment in cervical IDH patients have occasionally been reported, whether it is effective is yet a matter of controversy, and there is a paucity of studies on the effect of complementary and alternative medicine (CAM) treatment.

According to Benefits by Frequency of Disease data from the 2013 Korean National Health Insurance Statistical Yearbook [21], 5585 patients received treatment for cervical disc disorders for 99,582 days in outpatient care, of which 100,205 days were covered by the National Health Insurance, and medical treatment expenses eligible for reimbursement surmounted to 5,370,217 Korean Won, with 4,004,731 Korean Won reimbursed. Cervical disc disorders was the 12th most frequent reason for admission to Korean medicine hospitals, showing that it is not uncommon to receive inpatient care for cervical IDH.

Such CAM treatments as acupuncture, pharmacopuncture, herbal medicine, and manual therapy are well-sought in Korea to the aim of securing a less invasive, non-surgical method of treatment. Jaseng Hospital of Korean medicine, a Korean medicine hospital accredited by the Korean Ministry of Health and Welfare to specialize in spine disorders, treats over 900,000 spinal disease outpatient cases per year. This hospital manages patients with an integrative system utilizing conventional and Korean medicine, where conventional doctors and Korean medicine doctors (KMDs) cooperate for optimal treatment results. Conventional doctors participate in diagnosis using imaging technology such as X-rays and MRIs, and in treatment by caring for a small percentage of patients potentially in need of more intensive care. KMDs supervise and manage the main treatment of all patients, and decide whether the patient requires additional diagnosis and treatment from a conventional doctor. Cervical IDH patients suffering neck pain or radiating pain unable to receive outpatient treatment are thus provided with concentrated non-surgical integrative treatment during admission.

Despite the widespread use of inpatient treatment for cervical IDH encompassing a number of treatment modalities, studies on its treatment effect in patients admitted for cervical IDH are scarce. An integrative inpatient treatment approach with focus on CAM may not be widely available to patients, and the objective of this study is to introduce and assess the feasibility and long term effect of this integrative treatment model in inpatients with cervical IDH using a practical study design.

## Methods

### Study design

This study is a prospective observational study. We observed patients with a main complaint of neck pain or radiating arm pain diagnosed as cervical IDH and admitted from January 2011 to September 2014 at Jaseng Hospital of Korean medicine in Korea which provides integrated conventional and Korean medicine services with CAM as the main modality. The authors conducted a long term follow-up by phone interview during March 2015. Outcome measures covered 5 parts: numeric rating scale (NRS), neck disability index (NDI), patient global impression of change (PGIC), ever-surgery after discharge, and current treatment.

This study is a report on part of a registry collecting prospective data on integrated treatment for musculoskeletal disorder patients (ClinicalTrials.gov Identifier: NCT02257723). The study protocol was approved by the Institutional Review Boards of Jaseng Hospital of Korean medicine. All participants gave written informed consent prior to participation.

### Participants

Patients meeting the following criteria were included.

1. Admission for treatment of neck pain or radiating arm pain
2. Cervical IDH confirmed on MRI
3. Diagnosis by KMD that main cause of chief complaint (neck pain or radiating pain) is cervical IDH

Patients meeting the following criteria were excluded.

1. Main complaint other than neck pain or radiating pain
2. Concomitant musculoskeletal complaint (e.g. low back pain, knee pain)
3. Cause of neck pain unrelated to cervical IDH (e.g. spinal tumor, pregnancy, rheumatoid arthritis).
4. Refusal to participate in the study or nonagreement to collection and disclosure of personal information for study purposes

KMDs assessed the cause of current neck pain or arm pain symptoms with reference to neurological test results (sensory loss, motor weakness, and tendon reflex) and MRI readings by radiology specialists. Patients who met the proposed inclusion criteria were visited at the inpatient ward on the first day of admission for assessment by a KMD, and followed up using a similar interview and survey process upon discharge. If a patient was admitted multiple times during the study period, only the first admission record was appraised and included.

### Interventions

Though the treatment protocol was comprised with most frequented treatments for cervical IDH patients, any and all treatment methods not included in the treatment protocol were allowed and available to all physicians and patients and use of these treatments (type and frequency) were recorded in electronic medical records pragmatically. Conventional treatments such as pain medications and epidural injections (using local anesthetics such as lidocaine, steroids, and anti-adhesion adjuvants) were administered by a conventional rehabilitation specialist through KMD referral. Only non-surgical treatments were allowed during admission.

### Complementary and alternative medicine treatment protocol

Herbal medicine was taken 3 times/day in pill (2 g) and water-based decoction form (120 ml) (*Ostericum koreanum*, *Eucommia ulmoides*, *Acanthopanax sessiliflorus*, *Achyranthes bidentata*, *Psoralea corylifolia*, *Saposhnikovia divaricata*, *Cibotium barometz*, *Lycium chinense*, *Boschniakia rossica*, *Cuscuta chinensis*, *Glycine max*, and *Atractylodes japonica*). These herbs were carefully selected from herbs frequently prescribed for IDH treatment in Traditional Chinese Medicine and Korean Medicine [22] and the prescription was further developed through clinical practice [23]. The main ingredients of the herbal medicine used in this study (*Acanthopanax sessiliflorus* Seem, *Achyranthes japonica* Nakai, *Saposhnikovia divaricata* Schischk, *Cibotium barometz* J. Smith, *Glycine max* Merrill, and *Eucommia ulmoides* Oliver) have been studied in vivo and in vitro as GCSB-5 for their anti-inflammatory [24], and nerve [25] and joint protective effects [26], and clinically for non-inferiority in safety and efficacy compared to Celecoxib in treatment of osteoarthritis [27].

Acupuncture was administered 1–2 sessions/day at cervical Ah-shi points and acupuncture points pertaining to neck pain. Ah-shi point acupuncture refers to acupuncture needling of painful or pathological sites. Ah-shi points do not exactly match tender points or Buding, Tianying points, but generally correspond to points that induce relaxation or pain upon palpation [28].

The pharmacopuncture solution was prepared with ingredients similar to the orally administered herbal medicine (*Ostericum koreanum*, *Eucommia ulmoides*, *Acanthopanax sessiliflorus*, *Achyranthes bidentata*, *Psoralea corylifolia*, *Saposhnikovia divaricata*, *Cibotium barometz*, *Lycium chinense*, *Boschniakia rossica*, *Cuscuta chinensis*, *Glycine max*, and *Atractylodes japonica*) by decocting and freeze drying, then mixing the prepared powder with normal saline and adjusting for acidity and pH. Pharmacopuncture was administered 1 session/day at cervical Hyeopcheok (Huatuo Jiaji, EX B2) and Ah-shi points up to 1 cc using disposable injection needles (CPL, 1 cc, 26G x 1.5 syringe, Shinchang medical co. Korea).

Bee-venom pharmacopuncture was applied if the skin reaction test to bee-venom was negative. Diluted bee-venom solution (mixed with normal saline at a ratio of 1000:1) was injected at 4–5 cervical Hyeopcheok (Huatuo Jiaji, EX B2) and Ah-shi points at the physician’s discretion. Each point was injected with about 0.2 cc up to a total 0.5–1 cc using disposable injection needles (CPL, 1 cc, 26G x 1.5 syringe, Shinchang medical co. Korea)

Chuna spinal manipulation [29, 30], which is a Korean manipulation method that combines conventional manipulation techniques with high-velocity, low amplitude thrusts to joints slightly beyond the passive range of motion, and manual force within the passive range, was conducted 3–5 sessions/week.

### Outcome measures

All outcomes were assessed by KMDs who had received prior training and education. Demographic and health behavior characteristics (sex, age, occupation, smoking, alcohol consumption, and underlying disease) were collected on the first day of admission using short surveys on current pain levels and neurological exams. Follow-ups were conducted at 2 weeks post-admission or upon discharge and after discharge.

NRS [31] uses an 11-point scale to evaluate current neck pain and radiating pain where no pain is indicated by ‘0’, and the worst pain imaginable by ‘10’. NRS was assessed at admission, discharge, and long term follow-up. Due to lack of references on minimum clinically important difference (MCID) of neck pain or radiating pain for NRS, MCID for visual analogue scale (VAS) was used for further evaluation of NRS.

The NDI [32] is a 10-item survey that assesses the degree of disability from 0 to 5 in fulfilling daily activities. The total is divided by 50, then multiplied by 100. NDI was assessed at admission and discharge.

PGIC [33] was used to assess patient satisfaction rate of current state after admission. Satisfaction was rated with a 5-point scale ranging from very satisfactory, satisfactory, slightly satisfactory, dissatisfactory, and very dissatisfactory at discharge and long term follow-up.

Participants underwent physical and neurological examination at admission and discharge for objective motor and sensory evaluation of the cervical region. Range of motion (ROM) for neck flexion and extension, distraction, compression, Valsalva, Spurling, Adson’s, and swallowing tests, and upper extremity motor strength and sensory tests and deep tendon reflex tests were performed.

### Safety assessments

All potential adverse events regarding treatment, ranging from skin and local reactions to systemic reactions, and including change or aggravation in pain patterns were carefully observed, recorded and reported during admission. Adverse events associated with bee-venom therapy are known to range from skin reactions to severe immunological responses, and therefore adverse reactions including systemic immunological reactions requiring additional treatment (e.g. antihistaminic agents) were closely monitored. . Blood cell count, liver and renal function tests, and inflammatory activity tests were conducted in all patients at admission, and if there was an abnormal finding requiring follow-up as assessed by KMDs and conventional doctors, relevant markers were rechecked. A total 46 patients were judged to require follow-up at admission by KMDs and conventional doctors and were followed up accordingly during hospital stay, of which 9 patients showed abnormal findings in liver function at admission. Liver function was tracked in these nine patients. Presence of liver injury was also measured to assess possibility of drug-induced liver injury from herbal or conventional medicine intake using a definition of (a) ALT or DB increase of 2× or over the upper limit of normal (ULN) or (b) combined AST, ALP, and TB increase, provided one of them is above 2 × ULN.

### Statistical methods

All analyses were conducted using statistical package SAS version 9.3 (SAS Institute, Cary, NC, USA), and *p* < 0.05 was regarded to be statistically significant. Continuous data is presented as mean and standard deviation, and categorical data as frequency and percent (%). The mean difference in NRS of neck pain, NRS of radiating pain, and NDI between admission (baseline), discharge and long term follow-up was analyzed for significance with 95 % confidence intervals (CIs). Satisfaction rate assessed with a 5-point Likert scale at long term follow-up was recategorized into binary values of satisfactory (very satisfactory, or satisfactory) and dissatisfactory (slightly satisfactory, dissatisfactory, and very dissatisfactory). Multivariable logistic regression analysis was conducted to calculate odds ratios (ORs) and 95 % CIs, and estimate the influence of predictive factors on satisfaction rate. Baseline factors that met *p* < 0.10 in univariate analysis were included in the final model with age and sex, and factors were selected using stepwise method (*p* < 0.05).

## Results

**Fig. 1:**
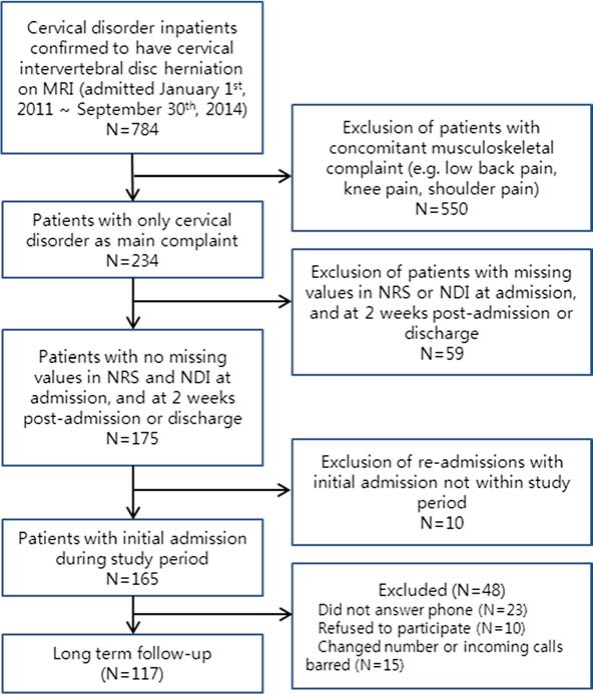
Flow diagram of the study

During the study period 784 patients with neck disorders were admitted, and of these, 234 patients were diagnosed with cervical IDH with no other major musculoskeletal complaints. Of the 234 cervical IDH patients, 175 patients had no missing values in NRS and NDI at admission and at 2 weeks post-admission or at discharge (short term follow-up). Ten patients were re-admissions and after inclusion of initial admission data if initial admission was during the study period, 165 patients remained. Long term follow-up assessments were conducted in 117 patients. In the non-long term follow-up group (*n* = 48), 23 patients did not answer the phone, 10 refused to participate in the long term follow-up, and 15 had since changed number or had incoming calls barred (Fig. 1). Baseline characteristics by long term follow-up group and non-long term follow-up group are listed in Table 1. Though there were no other marked differences between the 2 groups, 29 patients in the long term follow-up group had been recommended surgery (24.8 %), while only 1 patient in the non-long term follow-up group (0.02 %) had been recommended.

**Table 1:** Baseline demographic characteristics

Variables	Long term follow-up group (*n* = 117)		Non-long term follow-up group (*n* = 48)	
	n (%)	mean (sd)	n (%)	mean (sd)
Age (years)		45.4 ± 11.4		41.5 ± 10.4
Gender, male	54 (46.2)		25 (52.1)	
Smoking status				
Non-smoker	62 (53.5)		24 (50.0)	
Current smoker	33 (28.5)		14 (29.2)	
Past smoker	21 (18.1)		10 (20.8)	
Drinking				
No	14 (12.1)		3 (6.3)	
Yes	102 (87.9)		45 (93.8)	
Regular physical activity				
No	52 (44.8)		21 (43.8)	
Yes	64 (55.2)		27 (56.3)	
Body mass index		22.8 ± 3		22.8 ± 3.1
Previous neck pain episode				
No	93 (79.5)		40 (83.3)	
Yes	24 (20.5)		8 (16.7)	
Comorbid illness^a^, yes	65 (55.6)		27 (56.3)	
Radiating arm pain				
None	24 (20.7)		8 (16.7)	
Unilateral	67 (57.8)		29 (60.4)	
Bilateral	25 (21.6)		11 (22.9)	
Sensory loss	32 (42.7)		17 (58.6)	
ROM restriction^b^				
No	58 (49.6)		27 (56.3)	
Yes	59 (50.4)		21 (43.8)	
Positive physical exam result^c^				
No	31 (26.5)		19 (39.6)	
Yes	86 (73.5)		29 (60.4)	
Previous treatment for current episode				
Epidural injections^d^	19 (16.4)		6 (12.5)	
Analgesic medications	56 (48.3)		21 (43.8)	
CAM	25 (21.6)		13 (27.1)	
Surgery	1 (0.9)		2 (4.2)	
Duration of current episode (days)		210.1 ± 404.9		302.4 ± 557.6
Less than 1 month	34 (29.6)		11 (22.9)	
Between 1 and 6 months	54 (47.0)		22 (45.8)	
Over 6 months	27 (23.5)		15 (31.3)	
Recommendation for surgery at other institution^e^				
No	88 (75.2)		-	
Yes	29 (24.8)		1 (100)	
NRS, neck pain (0–10)		5.9 ± 2.3		5.8 ± 2.4
NRS, radiating arm pain (0–10)		4.8 ± 3.1		5.4 ± 2.7
Neck disability index (0–100)		40.6 ± 15.9		40.2 ± 15.2

*ROM* Range of motion; *CAM* Complementary and alternative medicine; *NRS* Numeric rating scale

^a^Any self-reported gastritis, tuberculosis, poliomyelitis, cardiovascular disease, uterine myoma, or hepatitis B carrier

^b^Regarded no ROM restriction if (a) cervical flexion > 30^o^ at admission, (b) cervical extension > 30^o^ at admission, (c) left cervical rotation angle = right cervical rotation angle at admission, and (d) left cervical side-bending angle = right cervical side-bending angle at admission were all satisfied. Regarded ROM restriction if any of the above-mentioned conditions were not met

^c^Regarded positive physical exam result if any exam of (a) compression test, (b) distraction test, (c) Valsalva test, (d) Spurling test was positive at admission. Regarded negative physical exam result if all exams were negative. If any exam was uncheckable due to pain or functional disability, it was considered to be a positive test result

^d^Local anesthetics such as lidocaine, steroids, and anti-adhesion adjuvants were used

^e^Ever-recommendation for surgery at other conventional institution before current admission

Average length of hospital stay was 20.8 ± 11.2 days. The majority of participants received inpatient treatment focused on Korean medicine and CAM. Herbal medicine was taken in accordance with the treatment protocol in decoction form by 81.8 % of patients and in pill form in 86.1 %, and the other patients were prescribed other herbal medicines at the KMD’s discretion. In use of conventional treatments not specified in the CAM treatment protocol, 18.2 % patients took analgesic medications or intramuscular injections an average 2.7 ± 2.3 times, and 4.8 % patients were administered 1.6 ± 0.5 epidural injections during hospital stay (Table 2). We did not implement restrictions in pharmacological treatment for study purposes, and allowed conventional medicine physicians full freedom to assess and prescribe conventional medicine as the physician deemed necessary for the patient. NSAIDs, antidepressants, and muscle relaxants were the main medicines used, and opioids were administered in the short-term in only 2 patients.

**Table 2:** Length of hospital stay and interventions administered during stay

Variables	Long term follow-up group (*n* = 117)		Non-long term follow-up group (*n* = 48)	
	n (%)	mean (sd)	n (%)	mean (sd)
Length of hospital stay (days)		22.1 ± 12.0		17.5 ± 8.4
Complementary and alternative medicine				
Herbal medicine^a^	117 (100)	180.7 ± 127.1	47 (97.9)	174.8 ± 117.4
Protocol decoction	94 (80.3)	61.9 ± 41.8	41 (85.4)	72.2 ± 44.3
Protocol pills	100 (85.5)	89.9 ± 55.3	42 (87.5)	86.9 ± 54.6
Acupuncture	117 (100)	33.8 ± 18.7	48 (100)	26.0 ± 13.7
Electroacupuncture	104 (88.9)	20.9 ± 15.6	44 (91.7)	15.4 ± 11.2
Pharmacopuncture	117 (100)	23.0 ± 12.9	48 (100)	17.8 ± 9.7
Bee venom pharmacopuncture	50 (42.7)	19.7 ± 10.1	23 (47.9)	14.2 ± 8.4
Chuna manipulation	98 (83.8)	12.7 ± 7.5	38 (79.2)	10.6 ± 6.1
Conventional treatment				
Analgesic medications	19 (16.2)	3.1 ± 2.7	11 (22.9)	2.1 ± 1.4
Epidural injections^b^	6 (5.1)	1.5 ± 0.5	2 (4.3)	2

^a^Herbal medicine protocol: A standardized herbal medicine prescription was recommended for all patients prior to commencement of study. However, the protocol allowed for individual tailoring according to patient characteristics and clinical symptoms as deemed necessary by KMDs

^b^Local anesthetics such as lidocaine, steroids, and anti-adhesion adjuvants were used

NRS of neck pain, NRS of radiating pain, and NDI all decreased significantly at discharge and at long term follow-up compared to baseline (admission) (Table 3). The major site of pain of neck and radiating arm pain showed a decrease larger than MCID (NRS decrease of 2.5 or larger in neck pain or radiating pain), and NDI scores also improved over the MCID score of 7.5 [34, 35]. Difference in NRS at discharge in the long term follow-up group (*n* = 117) was 2.71 (95 % CI, 2.33, 3.09) for neck pain, 2.33 (95 % CI, 1.9, 2.77) for arm pain, and that of NDI, 14.6 (95 % CI, 11.89, 17.32). Difference in NRS at long term follow-up for neck pain and arm pain from baseline was 3.15 (95 % CI, 2.67, 3.64) and 2.64 (95 % CI, 1.99, 3.29), respectively. Difference in NRS at discharge in the non-long term follow-up group (*n* = 48) was 2.83 (95 % CI, 2.22, 3.45) for neck pain, 2.48 for arm pain (95 % CI, 1.84, 3.12), and that of NDI was 14.86 (95 % CI, 10.41, 19.3). The between-group difference in effect between admission and discharge in the long term follow-up and non-long term follow-up patients was not significant (NRS of neck pain : *p*-value = 0.741; NRS of radiating arm pain: *p*-value = 0.646; Neck disability index: *p*-value = 0.775).

**Table 3:** Comparison of numeric rating scale for neck and radiating arm pain and neck disability index score in long term follow-up group and non-long term follow-up group

	Long term follow-up group (*n* = 117)			Non-long term follow-up group (*n* = 48)	
	Baseline (admission)	Short term follow-up (discharge)	Long term follow-up	Baseline (admission)	Short term follow-up (discharge)
NRS, neck pain	5.9 ± 2.29	3.19 ± 2.08	2.74 ± 2.27	5.81 ± 2.4	2.98 ± 2.03
Diff (95 % CI)^a^		2.71 (2.33, 3.09)	3.15 (2.67, 3.64)		2.83 (2.22, 3.45)
NRS, radiating arm pain	4.8 ± 3.09	2.47 ± 2.09	2.16 ± 2.43	5.38 ± 2.69	2.9 ± 2.32
Diff (95 % CI)^a^		2.33 (1.9, 2.77)	2.64 (1.99, 3.29)		2.48 (1.84, 3.12)
Neck disability index	40.57 ± 15.94	25.96 ± 16.06	-	40.24 ± 15.18	25.38 ± 14.59
Diff (95 % CI)^a^		14.6 (11.89, 17.32)	-		14.86 (10.41, 19.3)

*NRS* Numeric rating scale

^a^Difference from baseline (95 % confidence interval)

The average period from admission to long term follow-up was 625.36 ± 196.7 days. All 165 patients answered the PGIC at discharge, and of these patients 84.2 % replied that their state was “satisfactory” or higher. A total 117 patients replied to PGIC at long term follow-up, and 79.5 % rated their current state to be “satisfactory” or higher. PGIC was reported to be very satisfactory in 48 patients (41.0 %), satisfactory in 45 (38.5 %), slightly satisfactory in 18 (15.4 %), and dissatisfactory in 6 (5.1 %). Nine patients had undergone surgery (7.6 %), while 21 patients replied that they were currently receiving treatment. Of patients currently under treatment, 10 patients (8.5 %) continued to receive CAM, 12 patients (10.3 %) had selected conventional treatment, and 1 patient was receiving both (Table 4).

**Table 4:** Period from admission date to long term follow-up, and patient global impression of change, ever-surgery and current treatment status in long term follow-up group

Variables	n (%) / mean (sd)
Period from admission date to long term follow-up (days)	625.36 ± 196.7
No. of admissions	
1 admission	108 (92.3)
2 admissions	5 (4.3)
3 admissions	4 (3.4)
PGIC at discharge	
Very satisfied	41 (38.3)
Satisfied	51 (47.7)
Slightly satisfied	12 (11.2)
Dissatisfied	3 (2.8)
Very dissatisfied	
PGIC at long term follow-up	
Very satisfied	48 (41.0)
Satisfied	45 (38.5)
Slightly satisfied	18 (15.4)
Dissatisfied	6 (5.1)
Very dissatisfied	-
Ever-surgery after discharge^a^	
No	156 (7.6)
Yes (1 surgery)	9 (92.4)
Current treatment^b^	
None	96 (82.1)
CAM	10 (8.5)
Conventional treatment	12 (10.3)

*PGIC* patient global impression of change; *CAM* Complementary and alternative medicine

^a^Ever-surgery referred to cervical operations undertaken between discharge and long term follow-up

^b^Current treatment included treatments received within a week previous to long term follow-up, and types were recategorized into CAM and conventional treatments

Sex, age, and unilateral radiating pain satisfied *p* < 0.10 in univariate analysis of baseline characteristics. Satisfaction rate increased with older age in multivariate analysis. Patients with unilateral radiating arm pain tended to be more satisfied with treatment that those without radiating pain. Also, patients receiving CAM treatment showed higher satisfaction rates than patients receiving no treatment (Table 5).

**Table 5:** Assessment of predictive baseline factors associated with satisfaction rate

N (Case)	Univariable		Multivariable	
117 (93) (ref. dissatisfied)	OR	95 % CI	OR	95 % CI
Age (continuous)	1.067	(1.02, 1.12)	1.066	(1.02, 1.12)
Gender, male (ref. female)	1.257	(0.51, 3.12)	1.093	(0.43, 2.81)
Smoking status				
Past smoker (ref. non-smoker)	2.520	(0.52, 12.24)		
Current smoker (ref. non-smoker)	0.707	(0.27, 1.89)		
Drinking (ref. no)	2.427	(0.73, 8.07)		
Body mass index (continuous)	1.054	(0.90, 1.23)		
Previous neck pain episode (ref. no pain)	0.543	(0.20, 1.52)		
Comorbid illness (ref. no comorbidity)^a^	1.325	(0.54, 3.26)		
Radiating arm pain				
Unilateral (ref. none)	2.654	(0.86, 8.18)	4.513	(1.2, 17.03)
Bilateral (ref. none)	0.875	(0.26, 2.96)	0.977	(0.24, 3.95)
ROM restriction (ref.no)^b^	1.938	(0.77, 4.87)		
Positive physical exam result (ref.no)^c^	1.522	(0.58, 4.02)		
Previous treatment for current episode				
Epidural injections (ref. no)	0.974	(0.29, 3.26)		
Analgesic medications (ref. no)	1.132	(0.46, 2.79)		
CAM (ref. no)	2.200	(0.60, 8.08)	8.793	(1.46, 52.97)
Duration of current episode (days)				
Between 1 and 6 months (ref. <1 mos.)	0.862	(0.26, 2.83)		
Over 6 months (ref. <1 mos.)	0.345	(0.10, 1.19)		

*OR* Odds ratio; *ROM* Range of motion; *CAM* Complementary and alternative medicine

Only statistically significant variables from univariate regression were included using stepwise method in multivariable logistic regression with age and gender (*p* < 0.05)

^a^Any self-reported gastritis, tuberculosis, poliomyelitis, cardiovascular disease, uterine myoma, or hepatitis B carrier

^b^Regarded no ROM restriction if (a) cervical flexion > 30^o^ at admission, (b) cervical extension > 30^o^ at admission, (c) left cervical rotation angle = right cervical rotation angle at admission, and (d) left cervical side-bending angle = right cervical side-bending angle at admission were all satisfied. Regarded ROM restriction if any of the above-mentioned conditions were not met

^c^Regarded positive physical exam result if any exam of (a) compression test, (b) distraction test, (c) Valsalva test, (d) Spurling test was positive at admission. Regarded negative physical exam result if all exams were negative. If any exam was uncheckable due to pain or functional disability, it was considered to be a positive test result

Liver function was measured in all patients at admission, and nine patients with liver enzyme abnormalities at admission received follow-up blood tests at discharge. Liver enzyme levels returned to normal in 6 patients at discharge, while 2 retained liver enzyme abnormalities, and 1 patient sustained liver injury and on further assessment was diagnosed with active hepatitis showing Hbs antigen positive and Hbs antibody negative. There were no cases of systemic immunological reactions to bee venom pharmacopuncture requiring additional treatment and no other adverse events were reported.

## Discussion

These results show that inpatient treatment primarily focused on CAM maintains long term effects of pain relief and functional improvement in cervical IDH patients with neck pain or radiating arm pain. NRS and NDI scores at discharge and at long term follow-up all displayed significant decrease. Also, as statistical significance and clinical significance may differ, we checked for MCID and confirmed that both NRS and NDI scores improved over MCID. MCID has been reported at 2.5 in VAS for neck pain and radiating arm pain, and 7.5 in NDI scores [34, 35]. Average improvement in pain and functionality scales all exceeded MCID, and these results are likely to be reflected in patient satisfaction rate. Out of 165 patients, 128 patients (84.2 %) rated their current state as “satisfactory” or higher at discharge. At long term follow-up, 9 (7.6 %) out of 117 patients were confirmed to have received neck surgery, and most patients showed continued decrease in NRS and NDI. In addition, 96 patients (82.1 %) currently did not receive treatment for neck pain symptoms, and 93 patients (79.5 %) replied their state was “satisfactory” or higher. As comparison of between-group difference in the long term follow-up and non-long term follow-up patients was not designed a priori, this data may be regarded to be a post hoc data analysis. The between-group difference in effect between admission and discharge in the long term follow-up and non-long term follow-up patients was not significant, and in MCID, which could be considered a more clinical measure, the 2 groups produced comparable results.

Despite the fact that all patients underwent intensive Korean medicine treatment for the duration of hospital stay, no adverse events related to treatment were reported, demonstrating the safety of integrative medicine with focus on CAM. The authors had previously conducted a retrospective study to assess safety of herbal medicine and combined intake of herbal and conventional medicine in liver function test results of 6894 inpatients hospitalized at Korean medicine hospitals, and test results of the cervical disc herniation patients included in the present study were also described [36].

A major strength of this study is that it depicts clinical practice and the results reflect treatment as it is actually practiced in Korea in conventional and Korean medicine integrative treatment settings focused on CAM. Protocol treatment was standardized and comprised of interventions whose efficacy has been confirmed in pilot studies and frequently used in clinical practice, but the protocol also allowed for individual tailoring according to patient characteristics and symptoms as seen necessary by KMDs, and the percentage and frequency of these deviations were recorded. The satisfaction rate assessed at discharge not only reflects patient attitude toward treatment effect, but also increased medical costs entailed by inclusion of various treatments. Taking into account that the participants of this study were not patients recruited through advertisements, but patients visiting a Korean medicine hospital from personal choice receiving no economic compensation for study participation, the fact that most patients’ satisfaction rate was high is particularly noteworthy. The results of this study contribute to an evidence base for superior efficacy of compositive treatment over individual treatment in patients diagnosed with cervical IDH, and verify feasibility of clinical implementation with consideration for increased compositive treatment costs.

The largest limitation of our study is probably the inherent quality of a prospective observational study lacking a control. We are unable to draw conclusions on whether the suggested CAM integrative treatment is superior to an active control (e.g. surgery, conventional non-surgical intervention) or the natural course of disease. Another limitation is the heterogeneity of the patient groups and treatment composition. Participants were cervical IDH patients of varying symptoms, severity and chronicity whose progress are generally known to differ, and interventions included conventional treatments such as epidural injections or pain medications in some cases. Therefore it would be more accurate to construe these results to be the effect of a conventional and Korean medicine integrative treatment system than that solely of CAM integrative treatment. The compliance rate of 74 % (*n* = 175) at 2 weeks post-admission or discharge out of 234 admitted patients is low, especially considering the short follow-up period. This low compliance may be related to patient attitude toward study participation. As participants did not receive direct compensation for trial participation, they may have lacked incentive to continue participation, and the possibility that patients who refused follow-up assessment were dissatisfied with admission treatment should be considered. Long term assessment was conducted by phone interview in 117 patients (70 %) out of 165 baseline participants partly due to lapse in time, which limited the amount and quality of long term information that could be gathered and led to further patient loss from loss of contact.

Another limitation is that we failed to conduct more comprehensive medical evaluations. For example, although participants were diagnosed as disc herniation to be the main pathology based on MRI readings and neurological symptoms by KMDs, additional imaging information such as pathological disc level and severity of herniation were not collected. Also, data on subsequent recurrences, duration of all episodes and whether some were absolutely cured were not included in long term follow-up assessments, limiting multidimensional evaluation. In addition, while these cervical IDH patients required admission for severe neck and arm pain and consequent functional disability, the fact that this was the first attack of neck pain for many may have been cause for more favorable outcome.

However, the influence of long term follow-up compliance may not be confined to availability but potentially be associated with long term treatment effectiveness. As difference in characteristics of long term follow-up and non-long term follow-up patients may be reflected in short-term outcomes assessed at discharge and types and amount of additional conventional treatment, the fact that this study did not consider for these potential effects through additional analyses is a further limitation of this study.

Controversy still surrounds the efficacy of treatments for cervical IDH. While epidural steroid injections are the commonest modality of conservative treatment used in the United States [37] various systematic reviews show that effects are highly variable and not conclusive [38–44]. Two approaches are widely used in epidural injections: interlaminar and transforaminal approaches. The transforaminal approach has been criticized for safety risks [45–50], and though safer than the transforaminal approach, the interlaminar approach also holds potential risks [51–56]. Reports on the efficacy of conventional medicine for neuropathic pain show conflicting results [57–61], and study results on physical therapy are also inconsistent [62–64].

Gebremariam et al. [65] evaluated the efficacy of various cervical IDH treatments in a recent review, and concluded that though the single published study on conservative treatment versus surgery showed that surgery led to better results than conservative treatment, lacking intergroup analysis, there is no evidence supporting that one treatment is more superior. Despite recommendations for initial conservative treatment and management, some patients may select surgery for cervical IDH to the main aim of alleviating radiating pain in neuropathy and preventing progression of neurological damage in myelopathy [66]. Although the evidence base of conventional conservative and surgical treatments for cervical IDH weighing the benefits and harms is somewhat insufficient, the area has been extensively studied, while there is a distinct paucity of correlative studies on CAM.

Manchikanti et al. [67] stated in a 2 year follow-up study comparing epidural injection treatment with lidocaine and a mix of lidocaine and steroids for cervical IDH that NRS in the lidocaine group was 7.9 ± 1.0 at baseline, and 3.8 ± 1.6 at the 2 year follow-up, while NRS in the lidocaine and steroid group was 7.9 ± 0.9 at baseline, and 3.8 ± 1.7 at the 2 year follow-up. NDI in the lidocaine group was 29.6 ± 5.3 at baseline, and 13.7 ± 5.7 at the 2 year follow-up, and NDI in the lidocaine and steroid group was 29.2 ± 6.1 at baseline, and 14.3 ± 6.9 at the 2 year follow-up. When compared to our study, though improvement in NRS is slightly bigger in the study by Manchikanti et al., that of NDI is similar. The baseline NRS was higher at 7.9 in this previous study, and they did not differentiate between neck pain and radiating pain in NRS assessment.

The 1 year follow-up results comparing conservative treatment and plasma disc decompression (PDD) for contained cervical IDH show that VAS scores decreased 65.73, while NDI decreased 16.7 in the PDD group (*n* = 61), and that VAS scores decreased 36.45, and NDI decreased 12.40 in the conservative treatment group (*n* = 57) [68]. However, the study subject was limited to contained cervical IDH, the outcome measure for pain was VAS preventing direct comparison, and the follow-up period was shorter than our study.

The model of integrative treatment used at a Korean medicine hospital may be highly disparate from CAM treatment models used in Western countries. Although CAM treatment is gaining widespread popularity in the West, CAM is usually limited to “complementary” rather than “alternative” medicine, and is generally practiced by conventional practitioners as an adjunctive to conventional treatment after education on acupuncture/naturopathy/etc. or through referral to CAM specialists, of whom some do not hold individual practice rights. On the other hand, Korea adopts a dual medical system where KMDs hold practice rights equal to conventional practitioners, and she does not employ a primarily family practice-based medical system, allowing patients the freedom of primary treatment selection of conventional treatment or Korean medicine treatment. The participants of this study were patients visiting and admitted to a Korean medicine hospital for Korean medicine treatment of cervical IDH, and the integrative treatment model implemented at this Korean medicine hospital does not use CAM as a supplementary measure. Therefore, treatment comprised of CAM treatment such as acupuncture, herbal medicine, Chuna manipulation, and bee-venom pharmacopuncture in most patients, and conventional treatment was administered by conventional doctors through referral in a select few. A total 18.2 % of patients received analgesic medications prescriptions 2.7 times over an average admission period of 20.8 days, which is equivalent to 1–2 days worth’s prescription (calculated as 2 times/day), and epidural injections were administered to only 4.8 %, which is low considering that these patients required admission. It can be surmised that the main objective of admission in conservative treatment for most cervical IDH patients is alleviation of pain. The fact that many inpatients displayed significant pain and functional recovery in this study holds relevance for patients considering selecting a Korean medicine hospital for conservative treatment over surgery. Also, patients were confirmed to have maintained their improved state at long term follow-up, and only 9 received surgery out of the 117 patients assessed in the long term.

Patients were divided into 2 groups by satisfaction rate as evaluated at long term follow-up with PGIC, and multivariable logistic regression analysis was conducted on baseline characteristics to assess predictive factors for satisfaction and dissatisfaction. Older age was associated with higher satisfaction rate, and unilateral radiating pain was shown to be related with higher satisfaction rates than no radiating pain. In addition, patients receiving CAM treatment were associated with higher satisfaction rates compared to those not receiving treatment. This could be partly explained by the fact that more older patients may have higher levels of pain and be in more advanced stages of degeneration, resulting in more favorable and satisfactory treatment outcomes. Similarly, patients with unilateral radiating pain suffer neurological symptoms likely to be more severe than those with no radiating pain. In addition, patients continuing to receive CAM treatment may be more favorably predisposed toward CAM, resulting in higher satisfaction rates.

While numerous prospective long term studies have been conducted on injection treatment or surgical procedures, those on CAM treatment and inpatient treatment are few. The results of this study are comparable to the prospective long term results of injection treatment. Few studies have been conducted on admission treatment for patients with a main complaint of cervical IDH, which may be related with the difference in general healthcare systems.

## Conclusions

In conclusion, although the observational nature of this study limits us from drawing more decisive conclusions lacking a control, 3 weeks’ integrative inpatient treatment mainly comprised of CAM applied to actual clinical settings may result in satisfactory results and pain and functional improvement maintained in the long term in neck pain or radiating arm pain patients diagnosed with cervical IDH.

## Abbreviations

IDH: Intervertebral disc herniation
CAM: Complementary and alternative medicine
KMD: Korean medicine doctor
NRS: Numeric rating scale
NDI: Neck disability index
PGIC: Patient global impression of change
MCID: Minimum clinically important difference
VAS: Visual analogue scale
ROM: Range of motion
ULN: Upper limit of normal
CI: Confidence interval
OR: Odds ratio
PDD: Plasma disc decompression
